# *Streptococcus intermedius* promotes synchronous multiple primary lung cancer progression through apoptosis regulation

**DOI:** 10.3389/fimmu.2024.1482084

**Published:** 2025-01-09

**Authors:** Yi Deng, Zhi Xiang Dong, Gao Hua Yang, William S. Krimsky, Yong Hang Tai, Hao Peng, Gui Ting Huang, Jia Xin Xu, Saiyad A. Sarkar, Jun Peng, Kai Qian

**Affiliations:** ^1^ Faculty of Life and Biotechnology, Kunming University of Science and Technology, Kunming, China; ^2^ The Affiliated Hospital of Kunming University of Science and Technology, Kunming, China; ^3^ Department of Pulmonary and Critical Care Medicine, The First People’s Hospital of Yunnan Province, Kunming, China; ^4^ Department of Thoracic Surgery, The First People’s Hospital of Yunnan Province, Kunming, China; ^5^ Department of Molecular and Clinical Medicine/Wallenberg Laboratory, Institute of Medicine, University of Gothenburg and Sahlgrenska University Hospital, Gothenburg, Sweden; ^6^ Gala Therapeutics, San Carlos, CA, United States; ^7^ School of Physics and Electronic Information, Yunnan Normal University, Kunming, China; ^8^ Department of Respiratory Medicine, Medstar Franklin Square Medical Center, Baltimore, MA, United States

**Keywords:** lung microbiota, synchronous multiple primary lung cancer, Streptococcus intermedius, cell cycle, apoptosis

## Abstract

**Background:**

Dysbiosis of the lung microbiome can contribute to the initiation and progression of lung cancer. Synchronous multiple primary lung cancer (sMPLC) is an increasingly recognized subtype of lung cancer characterized by high morbidity, difficulties in early detection, poor prognosis, and substantial clinical challenges. However, the relationship between sMPLC pathogenesis and changes in the lung microbiome remains unclear.

**Methods:**

In this study, 16S rRNA sequencing was performed on clinical samples to analyze lung microbiome composition. Real-time quantitative PCR (qPCR) was used to quantify bacterial abundance in lung tissues. In addition, flow cytometry was conducted to evaluate cell cycle progression and apoptosis in lung tumor cells.

**Results:**

Clinical cohort studies demonstrated that sMPLC occurrence is associated with disturbances in the lung microbiome. Notably, Streptococcus intermedius was enriched in the lungs of sMPLC patients compared with non-tumor controls and accumulated preferentially in tumor tissues. *S. intermedius* shortened the cell cycle and inhibited apoptosis in lung cancer cells. Analyses of oral and gut microbiomes in different patient cohorts revealed a strong correlation between oral microbiome imbalances and lung microbiome composition in sMPLC patients.

**Conclusions:**

These findings characterize the lung microbiota in sMPLC and identify *S. intermedius* as a potentially influential bacterial strain. This study provides significant new insights into the diagnosis and treatment of sMPLC.

## Introduction

Lung cancer is the leading cause of cancer-related deaths globally and was the most frequently diagnosed cancer in 2022, accounting for nearly 2.5 million new cases. It also remains the primary cause of cancer mortality, with an estimated 1.8 million deaths, underscoring the critical need for focused research and intervention (1). In 2023, approximately 238,340 individuals (117,550 men and 120,790 women) are expected to be diagnosed with lung cancer, and 127,070 are projected to succumb to the disease. The role of airway microbiota in the initiation and development of lung cancer has been highlighted recently (2). Epidemiological investigations have consistently demonstrated a robust link between *Chlamydia pneumoniae* infection and the induction of chronic inflammation, which contributes to tumorigenesis in the lung (3). An *in vitro* study conducted by Tsay et al. in A549 human adenocarcinoma cells demonstrated that when these cells were being exposed directly to bacterial products extracted from lung cancer patients, there was an upregulation of genes involved in the PI3K pathway (4). In a mouse model, immune system-microbiota interactions were linked to the development of cancer; disrupted local microbiota triggered the proliferation and activation of lung γδ T cells via IL-1β and IL-23-dependent mechanisms. The activation of lung γδ T cells produces IL-17 that promotes neutrophil recruitment and inflammation within the tumor microenvironment (5). However, lung cancer encompasses a diverse range of subtypes, and our knowledge of the mechanisms underlying the interaction between lung microbiota and lung cancer is still severely lacking.

Synchronous multiple primary lung cancers (sMPLCs), which are increasingly recognized as a significant lung cancer category in clinical practice, refer to lung malignancies occurring simultaneously or within a 6-month interval (6). In an individual with sMPLC, various lung cancer types may exhibit unique genomic profiles (7). Through whole-exome sequencing and *in vitro* validation using a CRISPR-Cas9-based experimental workflow, Yu et al. demonstrated that each multicentric primary tumor in MSLC harbors unique oncogenic alterations likely driven by distinct molecular events (8). However, the pathogenesis of sMPLC is largely unknown. Patients diagnosed with sMPLC exhibit notably higher levels of tobacco exposure compared with healthy people, and their tumors are often characterized by independently arising mutations in the *TP53* and *KRAS* genes (9). The DNA methylation patterns and associated immune profiles of sMPLC and single primary lung cancers (SPLCs) are significantly different, underscoring the crucial roles of DNA methylation and immune profiles in the initiation and development of sMPLC (10). In addition to host-derived factors, the lung microbiome may also play a significant role in influencing cancer initiation and progression. Thus, altered lung microbiota is an alternative mechanism for the pathogenesis of sMPLC. Using advanced live tissue sampling techniques, investigators have identified significant differences in the microbial composition of tumor tissues and adjacent non-cancerous tissues within the same patient. In addition, specific microbial subgroups are enriched in patients with sMPLC, suggesting that the lung microbiota may play a crucial role in the initiation and progression of the disease (11).

This study aimed to determine the role of lung microbiota in the development of sMPLC. We recruited three distinct clinical patient cohorts: non-tumor, SPLCs, and sMPLCs. Distinct patterns in the load, richness, and variation of lung microbiota specific to sMPLC were identified. Through *in vitro* experiments and multi-omics studies, we demonstrated that *Streptococcus intermedius* is significantly enriched in the lungs of sMPLC patients in our cohort, accumulates in the tumor tissues, and inhibits apoptosis in tumor cells to promote the development of sMPLC. Additionally, sequencing analyses of oral and gut microbiota revealed potential origins of the lung carcinogenic bacteria in sMPLC patients from oral microbiota dysbiosis. These findings offer novel insights into the pathogenesis of sMPLC and highlight potential cellular and molecular targets for therapeutic intervention in sMPLC.

## Methods

### Patient cohort and characteristics

The basic criteria for defining sMPLC is based on the modifications done by Antakli (12, 13);. The particular sampling criteria align with those presented in prior publications (11). The study protocol was reviewed and approved by the Research Ethics Board of the First People’s Hospital of Yunnan Province [Reference No. 20200009]. This study was conducted following the Declaration of Helsinki. Written informed consent was obtained from all participants before participating in the study. All patients received general anesthesia before lung sample collection. Oral cavity microbiota were sampled before intubation. The specific sampling methodology aligns with the description provided in the prior publication (11).

Twenty-seven participants, comprising 8 non-tumor controls, 9 patients with sMPLC, and 10 patients with SPLC, provided self-collected stool samples following detailed printed instructions. Saliva samples were collected from 33 participants, comprising 11 non-tumor controls, 10 patients with sMPLC, and 12 patients with SPLC. To collect saliva samples, participants were instructed to take saliva samples as soon as they woke up without eating or brushing their teeth. The participants were asked to rinse their mouths with water and spit 3 ml of saliva into a 50 ml tube. Saliva samples were stored at −80°C until use (14).

### Bacterial abundance

RT-PCR amplification was performed to validate the abundance of each bacterial strain in tumor tissues (**Supplementary Table S3**). First, the extraction procedure of total RNA from each tissue sample was performed using TRIzol reagent (Invitrogen, USA) following the manufacturer’s protocol. Next, RNA concentration and quality will be assessed using the NanoDrop spectrophotometer and Agilent 2100 Bioanalyzer. The thermal cycling conditions were as follows: hybridization of random primers, 25°C for 10 min; cDNA synthesis, 42°C for 30 min; reverse transcriptase inactivation, 99°C for 5 min; and cooling, 4°C (15).

Real-time quantitative PCR reactions were performed using the Power SYBR Green master mix (ThermoFisher Scientific, Waltham, MA, USA) and an ABI Prism 7900HT Sequence Detection System (Applied Biosystems, ThermoFisher Scientific). The thermal cycling conditions were as follows: initial denaturation step, 95°C for 10 min; 50 cycles, 95°C for 15 s and 65°C for 1 min. The amplification specificity was confirmed by melting curve analysis.

### Sequencing and analysis of 16S rRNA

Total genomic DNA was extracted from 121 samples (including 61 BALF samples, 33 saliva samples, and 27 stool samples) using the TGuide S96 Magnetic Soil/Stool DNA Kit (Tiangen Biotech (Beijing) Co., Ltd.) according to the manufacturer’s instructions. The hypervariable region V3-V4 of the bacterial 16S rRNA gene was amplified with the following primer pairs: 338F: 5’- ACTCCTACGGGAGGCAGCA-3’ and 806R: 5’- GGACTACHVGGGTWTCTAAT-3’. PCR products were verified on an agarose gel and purified through the Omega DNA purification kit (Omega Inc., Norcross, GA, USA). The purified PCR products were collected, and the paired ends (2 × 250 bp) were performed on the Illumina Novaseq 6000 platform. Next, the raw data were analyzed and processed with reference to the previously published method (16–20).

### Lung and bacterial cell co-culture experiments

Bacteria were obtained from the Guangdong Institute of Microbiology, China, and cell lines were procured from Department of Pulmonary and Critical Care Medicine. Frozen stocks of human lung cancer cells (A549) were rapidly thawed in a 37°C water bath and resuspended in complete culture medium (RPMI1640 with 10% FBS and 1% penicillin-streptomycin for A549). The cells were centrifuged at 1000 rpm for 5 minutes, and the supernatant was discarded. The cells were resuspended and transferred to cell culture flasks. Then cells were incubated at 37°C in a 5% CO_2_ atmosphere. For bacterial culture, *S. intermedius* from a cryopreserved stock was rapidly thawed in a 37°C water bath. A loopful of bacterial culture was inoculated into BHI medium and incubated overnight in an anaerobic incubator at 37°C. Subsequently, in accordance with previous publications (21), the cell cycle and apoptosis following co-incubation of *S. intermedius* and A549 cell line will be assessed. In brief, co-incubation will be performed using a bacterial cell infection ratio of 1:100 (MOI), and flow cytometry will be utilized for determining the cell cycle and apoptosis.

### Library construction and sequencing

A549 cell samples will be collected using the aforementioned methods for assessing cell cycle and apoptosis. The control group will consist of A549 + PBS, while the treatment group will include samples co-incubated with *S. intermedius* and A549. Three replicates will be prepared for both treatment and control groups. The collected samples will be promptly flash-frozen in liquid nitrogen and RNA extraction will be carried out using a kit according to the manufacturer’s instructions, followed by concentration and quality assessment. High-quality RNA samples were used to construct sequencing libraries. The specific method for library construction is consistent with the method described previously (22).

Next, RNA-seq paired-end sequencing will be performed using the Illumina platform. Following sequencing, quality control will be conducted on the sequencing data to remove low-quality sequences. Subsequently, these high-quality sequences will be aligned to the genome of *S. intermedius*. Differential expression genes (DEGs) between the two groups of samples will be identified using the RPKM (reads per kilobase per million mapped reads) method to calculate transcript expression levels. Then, differential expression analysis will be conducted using edgeR (https://bioconductor.org/packages/release/bioc/html/edgeR.html). The criteria for selecting DEGs between the two groups are: log FC (logarithm fold-change) ≥ 2 and FDR (false-discovery rate) ≤ 0.05. Finally, these DEGs will be subjected to Kyoto Encyclopedia of Genes and Genomes (KEGG) pathway enrichment analysis.

### Statistical analysis

Data are presented as mean ± standard deviation (SD). The unpaired Student’s t-test was employed to assess differences between the two groups when applicable. Pathway enrichment analysis was carried out utilizing the Kyoto Encyclopedia of Genes and Genomes (KEGG) database. Statistical significance was defined as P < 0.05. GraphPad Prism 8.0 software (GraphPad Software, San Diego, CA) was utilized for all analyses.

## Results

### Study population

**Figure 1:**
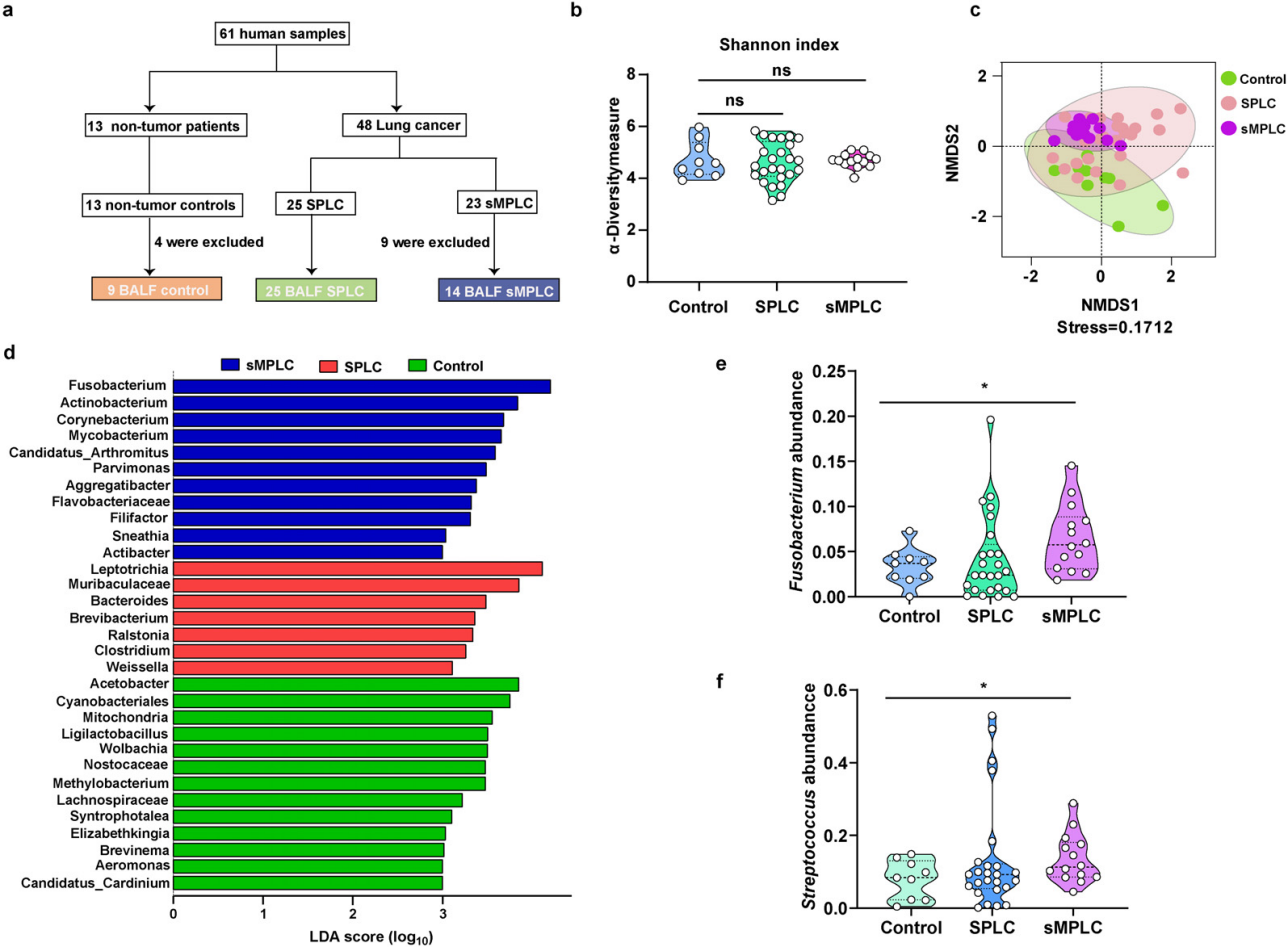
Marker gene sequence (16S rRNA) profiling of the lung microbiome in patients with sMPLC. **(A)** Patient enrolment: 61 patients participated in the study, comprising 13 non-tumor patients, 25 patients with SPLC, and 23 patients with SMPLC. However, four non-tumor patients and nine sMPLC patients were excluded from the cohort due to lack of patient consent or poor sample quality. **(B, C)** The α-diversity and β-diversity of lung microbiota from non-tumor patients (n = 9), patients with SPLC (n = 25), and patients with sMPLC (n = 14). **(D)** A plot of the linear discriminant analysis (LDA) scores from the LDA effect size analysis illustrating the differential abundance of the indicated taxa in the lung microbiomes of non-tumor patients and patients with SPLC (n = 25) and sMPLC (n = 14). **(E, F)** Statistical analysis of the relative abundance of Fusobacterium and Streptococcus. Data are presented as mean ± SD. Statistical significance was determined using unpaired Student’s t test. n.s., not significant; ∗p < 0.05.

Sixty-one volunteers were included in the study, comprising 13 non-tumor patients as the control group (3 cases removed due to quality control issues), 25 patients with single primary lung cancer (SPLC), and 23 cases patients with sMPLC patients (6 cases removed due to quality control issues), none of them have the history of smoking (**Figure 1A**). All patients underwent video-assisted thoracoscopic surgery. None of the participants had a history of smoking. Bronchoalveolar lavage (BAL) samples were obtained from all participants, as previously described (11).

To investigate the role of pulmonary microbiota in the initiation and development of sMPLC, BAL samples from all participants were sequenced using 16S rRNA. No significant changes in the α-diversity of the pulmonary microbiota in SPLC and sMPLC patients were detected compared with non-tumor patients (**Figure 1B**). However, the β-diversity of the pulmonary microbiota was significantly different in patients with SPLC and sMPLC compared with non-tumor patients (**Figure 1C**, stress < 0.2), suggesting a significant alteration in the pulmonary microbial ecology of SPLC and sMPLC patients. This result suggests that pulmonary microbiota dysregulation may play a crucial role in the occurrence and development of sMPLC.

### Lung microbiota characteristics

Venn analysis revealed that SPLC and non-tumor patients had 41 and 38 characteristic genera, respectively, while sMPLC patients had only three characteristic genera (**Supplementary Figure S1**, **Supplementary Table S1**). This result suggests that the composition of pulmonary microbiota is severely imbalanced in patients with sMPLC. Differential abundance analysis demonstrated enrichment of significantly different genera in the three cohorts (**Figure 1D**). *Fusobacterium*, *Parvimonas*, and *Aggregatibacter* were enriched in patients with sMPLC, and the *Bacteroides* genus was enriched in patients with SPLC compared with non-tumor patients. These genera are known opportunistic pathogens that are closely associated with the development of various diseases (23).

To identify the bacterial genera that only increased in the sMPLC cohort, we screened each genus for specific changes using relative abundance analysis. No significant differences in the relative abundance of *Fusobacterium* and *Streptococcus* were detected between non-tumor patients and patients with SPLC; however, the relative abundances of both genera were significantly increased in patients with sMPLC (**Figures 1E, F**). These results suggest that *Fusobacterium* and *Streptococcus* may play important roles in the development of sMPLC.

**Figure 2:**
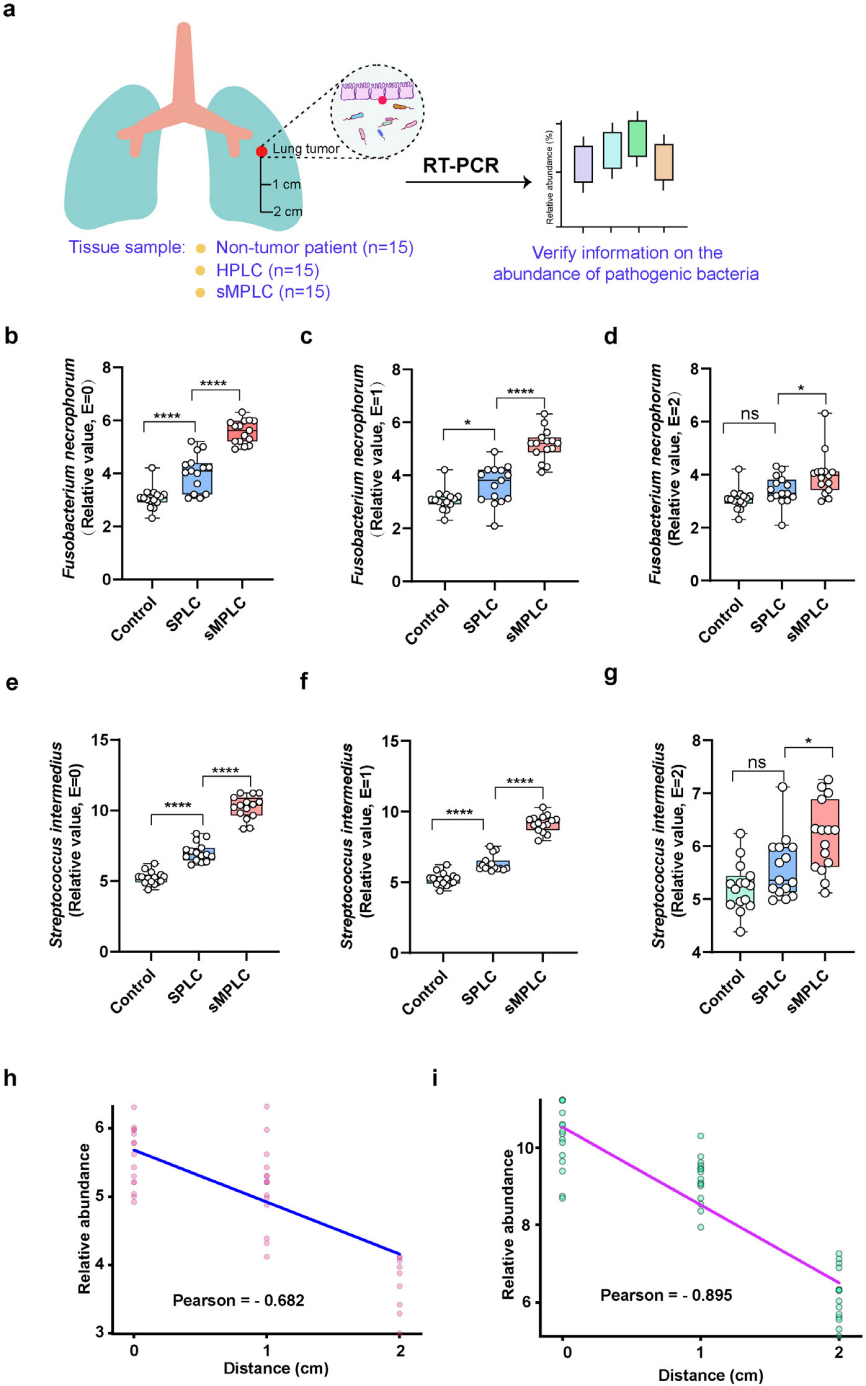
Correlation between lung microbiota and tumor types. **(A)** Sample collection characteristics and experimental flowchart. Normal tissue samples were collected from non-tumor patients (n = 15). Tissue samples were collected from the tumors and the surrounding tissue (1 and 2 cm from the tumor) in patients with SPLC and sMPLC (n = 15 per group). **(B–G)** The relative abundances of *Streptococcus intermedius* and *Fusobacterium necrophorum* were detected in the lung tissues using RT-PCR. **(H, I)** Pearson correlation was used to analyze the correlation between *S. intermedius* and *F. necrophorum* and the tumor distance. Data are presented as mean ± SD. Statistical significance was determined using unpaired Student’s t test (n = 15). n.s., not significant; ∗p < 0.05 and ∗∗∗∗p < 0.0001.

Next, the specific strain of *Fusobacterium* and *Streptococcus* that mediates the initiation and development of sMPLC was identified, and the potential differences in microbial abundance between tumor and paracancerous lung tissues were determined. Forty-five volunteers were recruited and categorized into non-tumor, SPLC, and sMPLC groups (15 patients per group) based on clinical indicators. Tissue samples were collected at 0 cm, 1 cm, and 2 cm from the tumor site (**Figure 2A**). In the control group, random normal tissue samples were collected. The 16S rRNA sequencing results revealed an increased relative abundance of bacteria, such as *Fusobacterium* and *Streptococcus*. Using RT-PCR, a significant distinction between *S. intermedius* and *Fusobacterium necrophorum* within the sMPLC group of conditionally pathogenic bacteria was identified. The relative abundances of *F. necrophorum* and *S. intermedius* were significantly higher in tumor tissues from patients with sMPLC than in tumor tissues from patients with SPLC and non-tumor tissues (**Figure 2B, E**). Despite a decrease in relative abundance with increasing distance from the tumor tissue, a similar trend persisted in tissues close (1 cm) to the tumor (**Figures 2C, F**). The relative abundances of *F. necrophorum* and *S. intermedius* in tissues 2 cm from the tumor were significantly higher in patients with sMPLC than in patients with SPLC and non-tumor tissues (**Figures 2D, G**). Furthermore, correlation analysis revealed that the closer the distance to the tumor tissue, the higher the relative abundances of *S. intermedius* and *F. necrophorum* (**Figures 2H, I**). The significant enrichment of *S. intermedius* and *F. necrophorum* in tumor tissues in sMPLC patients indicates their potential role in the development of sMPLC.

### *S. Intermedius* affects the apoptosis of lung tumor

**Figure 3:**
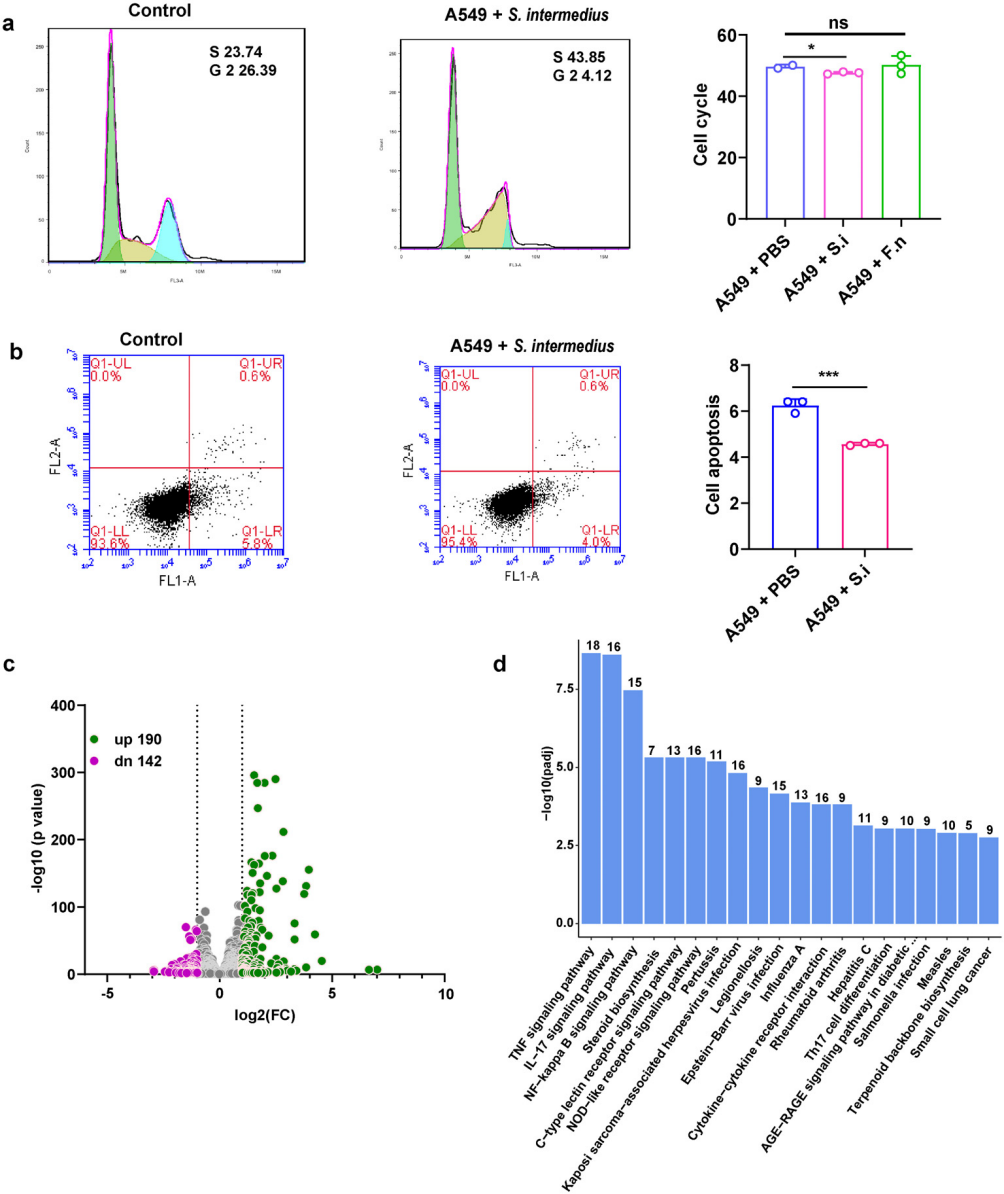
*Streptococcus intermedius* inhibits apoptosis in lung cancer cells (A549) to promote sMPLC development. **(A)** Flow cytometry analysis showing the inhibition of cell cycle progression in lung cancer cells in response to *S. intermedius*. *S. intermedius* (n = 3) and *F. necrophorum* (n = 3) were incubated with A549 cells, respectively, and the cell cycle was measured. **(B)** Flow cytometry analysis showing increased apoptosis in lung cancer cells in response to *S. intermedius*. *S. intermedius* (n = 3) was incubated with lung cancer cell line A549 cells and apoptosis was measured. The bacteria were cultured to logarithmic growth, collected and resuspended using sterile PBS. The control group was incubated with sterile PBS. **(C)** Comparative transcriptome analysis of A549 cells co-incubated with *S. intermedius*. The green symbols represent upregulated genes, and the purple symbols represent downregulated genes. **(D)** The enrichment of KEGG pathways in response to *S. intermedius*. The numbers above the bars represent the number of genes in the pathway. Data are presented as mean ± SD. Statistical significance was determined using unpaired Student’s t test. n.s., not significant; ∗p < 0.05 and ∗∗∗p < 0.001.

To understand how *S. intermedius* and *F. necrophorum* contribute to the development of sMPLC, the effects of the bacteria on cell cycle distribution in A549 lung cancer cells were determined using flow cytometry (**Figure 3A**). The cell cycle of A549 cells was significantly shorter in the presence of *S. intermedius* but not *F. necrophorum* (**Figure 3A**), and *S. intermedius* significantly reduced apoptosis in A549 (**Figure 3B**). These results show that *S. intermedius* promotes the growth of lung cancer cells by inhibiting apoptosis and activating the cell cycle process.

To determine the molecular pathways involved in the promotion of sMPLC by *S. intermedius*, transcriptome sequencing of A549 lung cancer cells was performed in the presence or absence of *S. intermedius*. *S. intermedius* induced the upregulation of 190 genes and the downregulation of 142 genes compared with untreated cells (**Figure 3C**). Importantly, pathways directly associated with lung tumors, such as small-cell lung cancer, were also significantly enriched (**Figure 3D**), supporting the role of *S. intermedius* in the development of lung tumors. KEGG enrichment analysis of the differentially regulated genes revealed the significant enrichment of 64 pathways, including the apoptosis pathway (**Supplemtary Table S2**), which confirms the effect of *S. intermedius* on apoptosis. In addition to apoptosis, several pathways related to inflammation and immune function were enriched, including the tumor necrosis factor (TNF), IL-17, and NF-kappa B signaling pathways and Th17 cell differentiation (**Figure 3D**). Thus, *S. intermedius* may affect the development of sMPLC by inducing changes in the immune microenvironment.

### Microbiota transmission via the oral-lung axis

**Figure 4:**
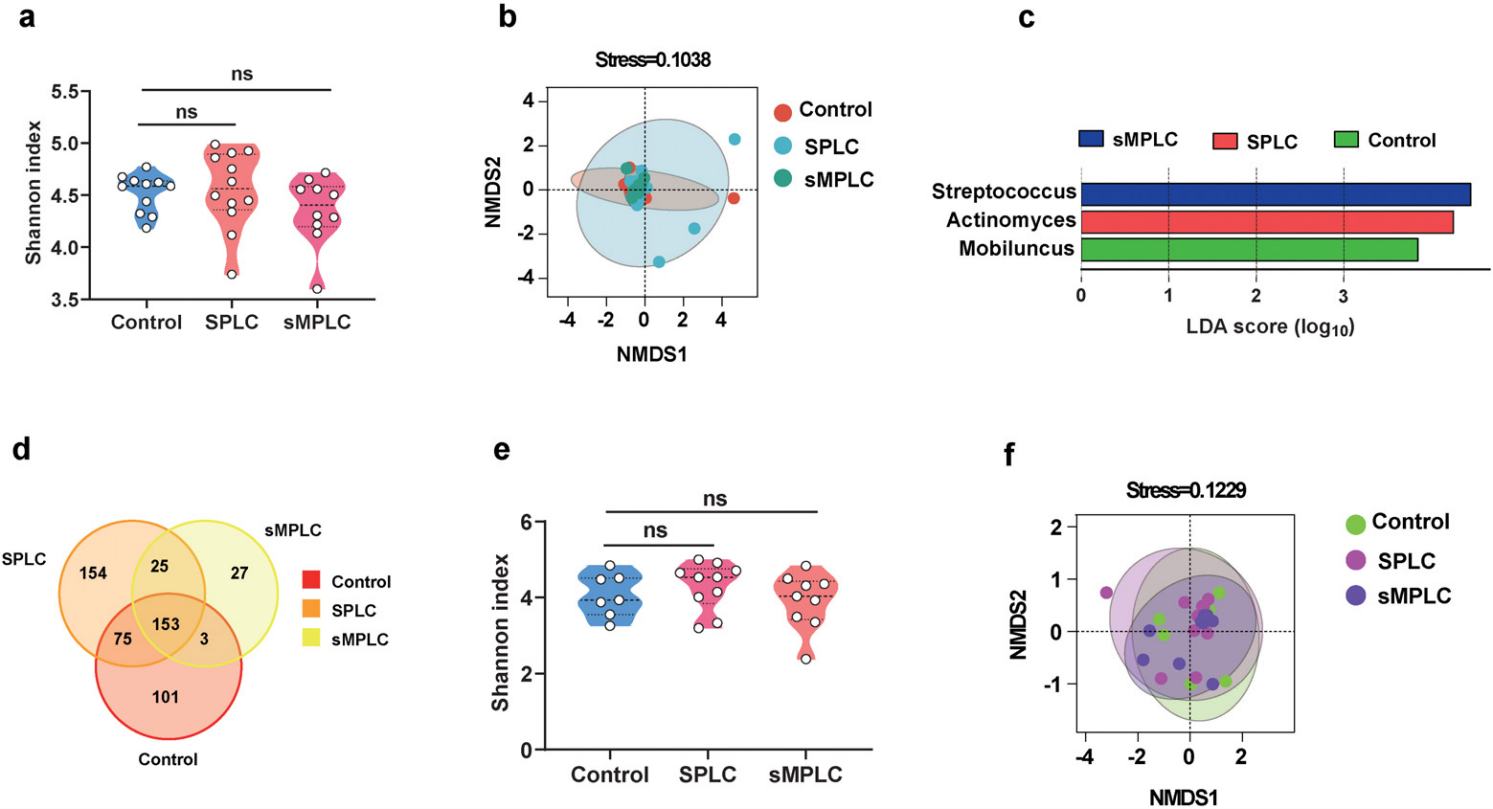
Disturbance of lung flora may be mediated by oral flora. **(A)** Analysis of the α-diversity of oral microbiota (Control = 11, SPLC = 12, sMPLC = 10). **(B)** The β-diversity of oral flora analyzed by DMDS. **(C)** A plot of linear discriminant analysis (LDA) scores from the LDA effect size analysis illustrating the differential abundance of the indicated taxa in the lung microbiomes of non-tumor patients and patients with SPLC and sMPLC. **(D)** Interactive analysis of the common and unique strains in the oral flora from non-tumor patients and patients with SPLC and sMPLC. **(E)** The α-diversity of gut microbiota (Control = 7, SPLC = 10, sMPLC = 9). **(F)** The β-diversity of gut microbiota (Control = 7, SPLC = 10, sMPLC = 9). Data are presented as mean ± SD. Statistical significance was determined using unpaired Student’s t test. n.s., not significant.

As dysregulation of lung microbiota contributes to the development of sMPLC, the sources of lung microbiota are important for the diagnosis and treatment of sMPLC. Thus, saliva and fecal samples collected from non-tumor patients and patients with SPLC and sMPLC were subjected to 16S rRNA sequencing. The α-diversity of oral microbiota in non-tumor patients, SPLC, and sMPLC did not change significantly (**Figure 4A**). However, the β-diversity of oral microbiota was significantly different in the sMPLC group compared with the non-tumor group (**Figure 4B**), indicating a substantial difference in the composition of oral microbiome profiles. Furthermore, linear discriminant analysis effect size revealed enrichment of *Streptococcus*, *Actinomyces*, and *Mobiluncus* in the oral microbiota of sMPLC, SPLC, and non-tumor patients, respectively (**Figure 4C**), and *Streptococcus* was significantly enriched in both the oral cavity and the lung tissue of patients with sMPLC as mentioned above. An intersection analysis revealed that the oral microbiota was similar to the lung microbiota in patients with sMPLC. As shown in **Figure 4D**, patients with sMPLC had fewer characteristic genera (only 27), whereas SPLC and non-tumor patients had 154 and 101 characteristic genera, respectively, indicating a significantly lower diversity of microbiota in the sMPLC group. These results highlight the close connection between the oral and lung microbiota.

Similar to the oral microbiota, the α-diversities of the gut microbiota were not significantly different between the three groups (**Figure 4E**), while the β-diversity showed significant differences (**Figure 4F**). However, we did not observe changes in *Streptococcus* in fecal microbiota. In addition, intersection analysis revealed that relatively few characteristic genera were detected in the fecal microbiota of patients with sMPLC, but the distribution of distinct characteristic microbiota between the groups was different from the oral and lung microbiota (**Supplementary Figure S2A**). Intersection analysis revealed 453, 137, and 83 characteristic genera in the gut, oral, and lung microbiota, respectively (**Supplementary Figure S2B**), and more microorganisms intersected in the oral-lung axis than in the lung-gut axis (**Supplementary Figure S2C**). In summary, these results suggest that *Streptococcus*, that lead to the development of sMPLC, may originate from the oral cavity.

## Discussion

The incidence of sMPLC has gradually increased due to the broader application of multislice spiral computed tomography, fluorescence endoscopy, and positron emission tomography (24). The precise pathogenic mechanism underlying multiple primary lung cancers remains elusive. In this study, we investigated the role of lung microbiota in the development of sMPLC. Our results demonstrate that lung microbiota in patients with sMPLC significantly differ from lung microbiota in patients without lung cancer or with single primary lung cancers. Of note, *S. intermedius* was significantly enriched in the sMPLC group and may contribute to or induce sMPLC.

The accumulation of *Streptococcus* is consistently linked with cancer progression. *Streptococcus gallolyticus* and *Streptococcus bovis* are strongly associated with colorectal cancer (25, 26), and *Streptococcus pneumoniae* plays an oncogenic role in the development and progression of lung cancer (27). In our study, *S. intermedius*, a common oral microbe, was more abundant in the tumor tissue of patients with sMPLC compared to adjacent non-tumorous lung tissues. The hypoxic microenvironment around tumor tissues may provide favorable conditions for the growth of facultative anaerobic bacteria, or the accumulation of tumor metabolites such as lactic acid may serve as substrates for bacterial metabolism. A previous study reported Fap2 and FadA synergistically contribute to the enrichment of *Fusobacterium nucleatum* in colorectal cancer (28), and specific receptors on the surface of lung tumor cells may also bind with *S. intermedius*, resulting in their enrichment.

To trace the origins of lung microbiome dysbiosis, oral and gut microbiota in the SPLC and sMPLC cohorts were analyzed. Consistent with findings from previous epidemiological studies, changes in the oral microbiome are highly associated with the risk of lung cancer (29). Notably, the number of characteristic species in the lung and oral microbiota in the sMPLC cohort was significantly lower than the number of characteristic species in the control and SPLC cohorts, indicating severe ecological disruption. A previous study also demonstrated the adverse effects of antibiotic treatment on cancer progression (30). The abundances of the *Streptococcus* genus in the lung and oral cavity were increased similarly, indicating that *S. intermedius* in the oral cavity may be a source of opportunistic pathogenic microbes in the lungs. Thus, oral intervention is a potential prevention strategy for sMPLC.

Co-culturing bacteria and tumor cells demonstrated that *S. intermedius* affects tumor development and initiation by inhibiting cell apoptosis, suggesting that the lung microbiome is a significant carcinogenic factor. Transcriptomic profile changes in cancer cells co-cultured with bacteria revealed significant alterations in several tumor and inflammation-related signaling pathways, including the TNF, IL-17, and NF-kappa B signaling pathways and Th17 cell differentiation. The TNF and IL-17 signaling pathways are upregulated in a chronic inflammatory environment, potentially promoting tumorigenesis (31, 32);. NF-κB enhances resistance to programmed cell death and inhibits the stimulation of gene expression for molecules in the p53 signaling pathway (33). Therefore, bacterial interference with the inflammation and apoptosis pathways could have significant implications for tumorigenesis and progression, although the detailed mechanism is still unclear. Deciphering these mechanisms is crucial to identifying new therapeutic targets in bacteria to aid in the prevention or treatment of cancer.

## Conclusions

In this study, the patients included in this study were primarily from Yunnan Province, China. As a result, the findings cannot be generalized to other populations. To gain a broader understanding of the bacterial-derived pathogenic mechanisms of sMPLC, it is essential to expand the cohort to include individuals from diverse regions and genetic backgrounds. Although our *in vitro* experiments confirmed the role of *S. intermedius* in promoting tumor cell growth, the precise biochemical mechanisms underlying this effect remain unclear. Future studies should focus on isolating bioactive compounds from *S. intermedius* or its metabolites to elucidate the molecular pathways involved in its anti-apoptotic effects. Such research could uncover potential molecular targets for the treatment of sMPLC. Moreover, based on the similarities observed between the oral and lung microbiomes, we hypothesize that *S. intermedius* originates from the oral cavity. Further metagenomic analyses, including single-nucleotide polymorphism (SNP) studies at the strain level, are needed to investigate the microbial transmission from the oral cavity to the lungs, which could provide valuable insights into the oral-lung microbiome axis. For high-risk populations, specialized oral hygiene management could be an effective strategy to reduce lung cancer incidence. In patients with lung tumors and concurrent microbiome dysbiosis, targeted therapies using pathogen-specific antibiotics or bacteriophage treatment may help reduce disease onset or slow tumor progression. These strategies offer promising avenues for improving prevention and treatment outcomes in lung cancer.

## Data Availability

The data presented in the study are deposited in the National Center for Biotechnology Information repository, accession number PRJNA1200823 and PRJNA1200287.

## Supplementary material

Supplementary Figure 1 Interactive analysis of the common and unique strains in the lung microbiota from non-tumor patients and patients with SPLC and sMPLC. The specific and unique strains of pulmonary flora in different groups were analyzed by Venn diagram.

Supplementary Figure 2 The oral-lung axis may mediate the spread of the flora. **(A)** Interactive analysis of the common and unique strains in the gut microbiota from non-tumor patients and patients with SPLC and sMPLC. **(B)** Interaction analysis of lung, gut and oral flora. **(C)** Interaction analysis of lung, gut and oral flora in patients with sMPLC.

Supplementary Table 1 Pulmonary strains unique or common to SPLC and sMPLC. This table shows the relative abundance of lung flora in different populations.

Supplementary Table 2 Pathways through which *S. intermedius* induces significant changes in tumor cells. Comparative transcriptome analysis of the major pathways affected by *S. intermedius* stimulation in lung cancer cells

Supplementary Table 3 Primers employed for RT-PCR experiments to detect bacterial strains.
