# Supplementary figures and images for: Streptococcus intermedius promotes synchronous multiple primary lung cancer progression through apoptosis regulation
Source: Front Immunol. 2025 Jan 9;15:1482084. doi: 10.3389/fimmu.2024.1482084 (PMC11754412; doi:10.3389/fimmu.2024.1482084)

**SPLC**

**sMPLC**

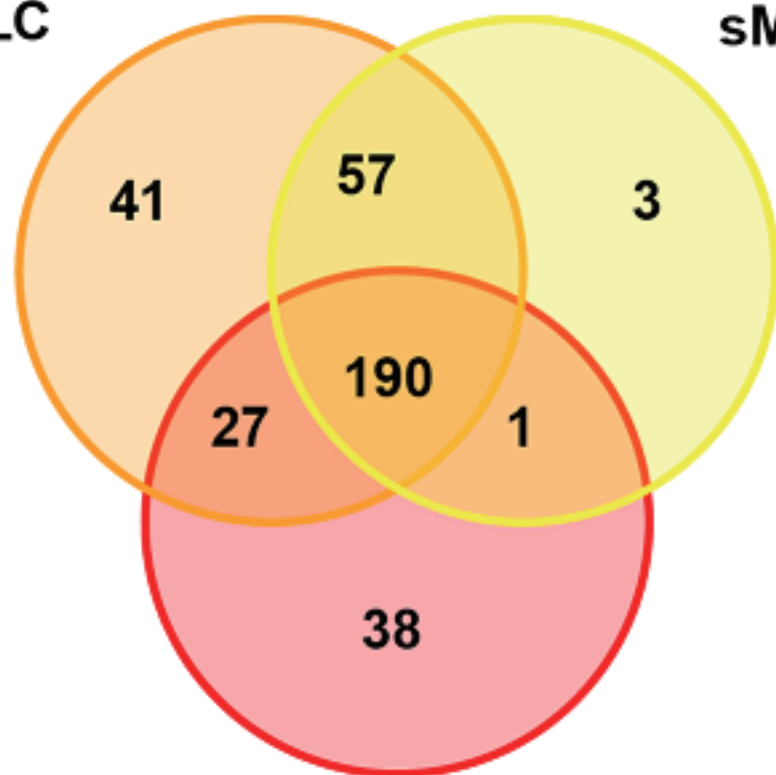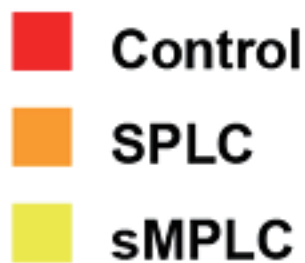

**Control**

Supplement: Supplementary Figure 1 — Interactive analysis of the common and unique strains in the lung microbiota from non-tumor patients and patients with SPLC and sMPLC. The specific and unique strains of pulmonary flora in different groups were analyzed by Venn diagram. [file Image1.pdf]

**a**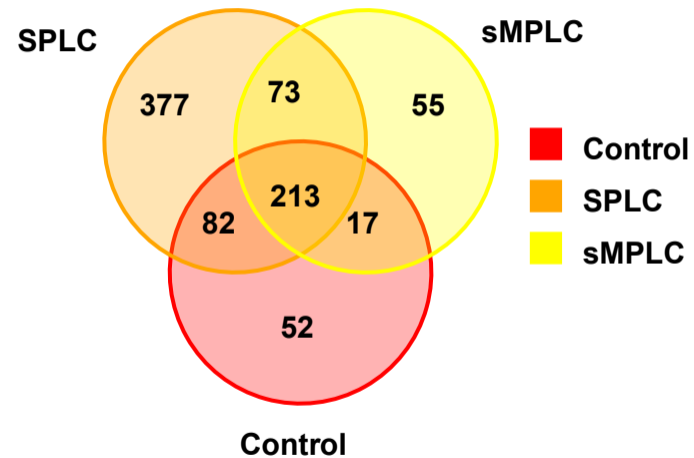**b**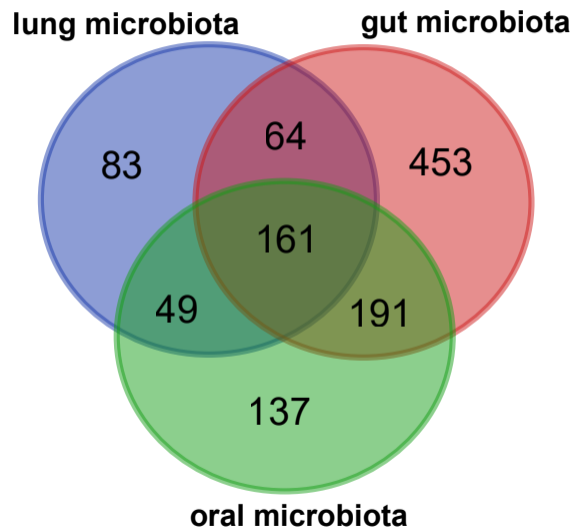**c**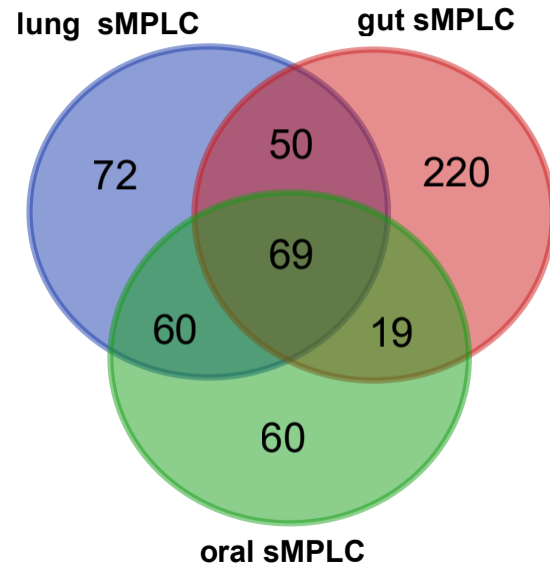

Supplement: Supplementary Figure 2 — The oral-lung axis may mediate the spread of the flora. (A) Interactive analysis of the common and unique strains in the gut microbiota from non-tumor patients and patients with SPLC and sMPLC. (B) Interaction analysis of lung, gut and oral flora. (C) Interaction analysis of lung, gut and oral flora in patients with sMPLC. [file Image2.pdf]
